# Lateral Flow Immunosensing of *Salmonella* Typhimurium Cells in Milk: Comparing Three Sequences of Interactions

**DOI:** 10.3390/microorganisms12122555

**Published:** 2024-12-11

**Authors:** Nadezhda A. Byzova, Irina V. Safenkova, Alexey A. Gorbatov, Sergey F. Biketov, Boris B. Dzantiev, Anatoly V. Zherdev

**Affiliations:** 1 A.N. Bach Institute of Biochemistry, Research Centre of Biotechnology of the Russian Academy of Sciences, 119071 Moscow, Russia; nbyzova@inbi.ras.ru (N.A.B.); safenkova@inbi.ras.ru (I.V.S.); dzantiev@inbi.ras.ru (B.B.D.); 2 State Research Center for Applied Microbiology & Biotechnology, 142279 Obolensk, Russia; gorbatov1986@mail.ru (A.A.G.); biketov@mail.ru (S.F.B.)

**Keywords:** *Salmonella*, food safety, immunochromatography, test strips, order of immunoreactants interactions

## Abstract

To ensure the safety of foodstuffs, widespread non-laboratory monitoring for pathogenic contaminants is in demand. A suitable technique for this purpose is lateral flow immunoassay (LFIA) which combines simplicity, rapidity, and productivity with specific immune detection. This study considered three developed formats of LFIA for *Salmonella* Typhimurium, a priority pathogenic contaminant of milk. Common sandwich LFIA with all immunoreagents pre-applied to the test strip (format A) was compared with incubation of the sample and (gold nanoparticle—antibody) conjugate, preceding the lateral flow processes (format B), and sequential passages of the sample and the conjugate along the test strip (format C). Under the chosen conditions, the detection limits and the assay times were 3 × 10^4^, 1 × 10^5^, and 3 × 10^5^ cells/mL, 10, 15, and 20 min for formats A, B, and C, respectively. The selected format A of LFIA was successfully applied to test milk samples. The sample’s dilution to a fat content of 1.0% causes pathogen detection, with 70–110% revealing and 1.5–8.5% accuracy. The obtained results demonstrate that the developed LFIA allows the detection of lower concentrations of *Salmonella* cells and, in this way, accelerates decision-making in food safety control.

## 1. Introduction

Despite the undeniable progress in food production technologies and safety control systems, foodborne diseases remain a serious threat to public health worldwide. A total of 600 million cases of foodborne diseases and 420,000 food production-related deaths are observed worldwide every year [1]. Milk and dairy products, which are actively consumed by people from all regions, with different ages and income levels, require serious attention as sources of pathogens entering the human body. The impact of dairy products on human foodborne diseases is estimated at 4–5% [2,3]. *Salmonella* spp., *Listeria monocytogenes*, *Campylobacter* spp., Shiga toxin-producing *Escherichia coli*, and coagulase-positive *Staphylococcus* spp. are stated as the main pathogens occurring in milk and milk products [4,5]. *Salmonella* serovars belong to this group and can be found in raw dairy products, affecting regions of both high and low economic levels, causing a significant impact on dairy-borne disease outbreaks [6,7]. In the USA alone, *Salmonella* causes about 1,350,000 infections and 26,500 hospitalizations every year [8].

The current situation is characterized by significant differences in the frequency of various *Salmonella* serovars in different types of food products [9] and in different regions [10]. For milk and dairy products, the most common contaminating serovar is *S*. Enteritidis, and *S*. Typhimurium typically takes second place. It is interesting to note that, in their investigation in England, Santos et al. found that *S*. Typhimurium requires higher costs for disease treatment compared to *S*. Enteritidis [11].

The existing regulations state extremely high requirements for controlled products, thus preventing the dangers of the further growth of an initial single bacterium at the final stages of trade and consuming chains. To confirm the accordance of tested food to zero tolerance standards stated by national and international authorities [12], the initially collected samples are subjects of selective cultivation for the detection of an increasing quantity of bacteria in case of their presence and, by this method, for making conclusions about the contamination of the tested food [13]. In classical assay formats, this growth is finalized by the formation of visible individual colonies on solid media that are then tested morphologically and biochemically for their accordance to the target bacteria [14]. However, such assays are extremely time-consuming; their implementation takes several days. The modern improved media for accelerated selective growth is combined with enzymatic reactions to accelerate assays, but this still requires many hours. Due to these alternate solutions, researchers are actively searching to achieve a possible broad control of foodstuffs for microbiological safety [15].

The significant progress that has been made in solving this task has been associated with the use of nucleic acid amplifications, such as polymerase chain reaction, loop-mediated isothermal amplification, and recombinase polymerase amplification, etc. These processes significantly accelerate reaching the final conclusion but cannot exclude the starting stage of bacterial growth. Considering the relatively rapid implementation of amplifications (0.5–2 h) and their detection limits (typically in the range of 10–100 cells/mL), the full testing cycle can be implemented in a few hours [16,17]. However, the significant limitations of these approaches are the necessity of sophisticated equipment and the sterility of working areas to exclude cross-contamination, and the involvement of qualified personnel. These factors limit the location of amplification-based techniques for food control by specialized laboratories.

In this collection, immunochemical assays seem to be effective solutions, especially lateral flow immunoassay (LFIA) that is maximally adapted for rapid on-site testing. LFIA may be implemented in a few (10–20) minutes with minimal pretreatment of samples; it is characterized by extremely simple and non-laborious protocols for the testing and evaluation of the obtained results [18,19,20]. Combining the steps of growing microorganisms from the collected food sample further and subsequent LFIA testing provides simple and easy control of pathogens. If even a single live bacterium gets into the sample during the growing stage, its quantity reaches the level that is detectable by LFIA. In this regard, improved LFIA techniques with lower detection limits are in demand, because they allow the growing time to be reduced, and they obtain the assay results faster. The recent efforts in the development of novel sensitive variants of LFIA are very variable [21,22,23,24,25]. Some of the propositions are associated with the use of new labels or the instrumental detection of alternative physical parameters [26,27,28], and so cannot be considered as ways for operating improvements of tests for wide screening. LFIA variants stored the most common gold nanoparticle (GNPs) labels, but with the changed procedure of immune complexes formation, the modified LFIAs seem more appropriate for this purpose. Recently, several variants with such a modulation of the order and rate of reactants interaction (pre-incubation, sequential flowing, etc.) were proposed, demonstrating improvements in sensitivity [29,30,31,32,33]. However, the universality of these approaches and their applicability to different immunoreagents remains unclear.

**Figure 1:**
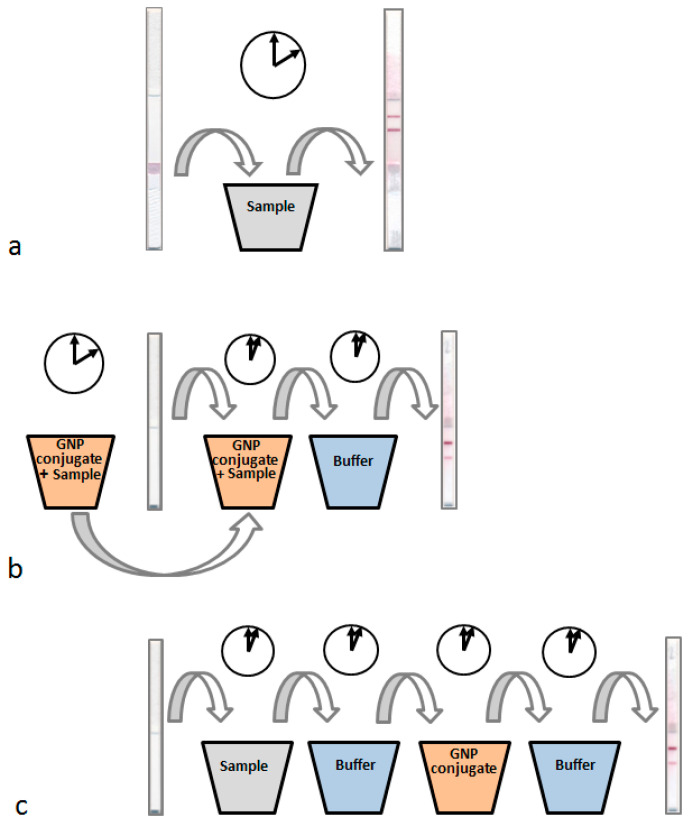
Three LFIA formats for *Salmonella* Typhimurium: format A (**a**), format B (**b**) and format C (**c**).

Based on the reasons given above, our study aimed for the development and comparative consideration of three LFIA formats for *Salmonella* Typhimurium (Figure 1): common sandwich LFIA with all immunoreagents pre-applied to the test strip (format A), LFIA with incubation of the sample and the (gold nanoparticle—antibody) conjugate, preceding the lateral flow processes (format B), and LFIA based on sequential passages of the sample and the conjugate along the test strip (format C).

The detection limits that were reached were interpreted based on the measured quantitative parameters of immune interactions, namely equilibrium and kinetic binding constants. Finally, the chosen assay format was characterized by its applicability for milk sample testing, including consideration of the efficient procedure for the samples’ pre-treatment.

Considering the choice of *S*. Typhimurium as the target analyte to be detected, it should be noted that the existing variety of available anti-*Salmonella* antibodies is characterized by very different specificities and the diverse coverage of priority serovars [34]. In this situation, the characterization and analytical application of antibodies that broaden the selectivity spectrum for recognizing *Salmonella* serovars appear to be useful, both as additional testing tools and as potential components for multiplex diagnostic tools. The interest in the detection of *S*. Typhimurium in milk is confirmed by the development of different types of immunodiagnostic systems such as electrochemical [35], fluorometric [36], chemiluminescent [37], and Mach–Zehnder interferometric [38] systems for this purpose.

## 2. Materials and Methods

### 2.1. Reagents and Materials

Monoclonal mouse antibodies against lipopolysaccharide (LPS) of *S.* Typhimurium were from HyTest (Moscow, Russia)—clone 1E6cc, and from the All-Russian Research Center of Molecular Diagnostics and Therapy (Moscow, Russia)—clones 10D9H, and 5D12A. Polyclonal goat anti-mouse immunoglobulins (GAMI) were from Arista Biologicals (Allentown, PA, USA). The GAMI, labeled by horseradish peroxidase (HRP), were from Imtek (Moscow, Russia). Streptavidin (STR) conjugated with HRP, bovine serum albumin (BSA), gold (III) chloride hydrate, Tris, Tween-20, Triton X-100, N-hydroxysuccinimide (NHS), N-hydroxysuccinimide ester of biotin, ethylenediaminetetraacetic acid (EDTA), 1-hydrochloride-ethyl-3-(3’-dimethylaminopropyl) carbodiimide (EDC), 3,3’,5,5’-tetramethylbenzidine (TMB), sucrose, sodium citrate, sodium azide, 30% hydrogen peroxide, and dimethyl sulfoxide (DMSO) were from Sigma-Aldrich (St. Louis, MO, USA). All other auxiliary chemicals (salts, acids, alkalis) were of analytical grade. Ultrapure water for all solutions was prepared using Milli-Q system from Millipore Corporation (Burlington, MA, USA) and had a conductivity of no more than 0.055 μS/cm at 25 °C.

The membranes for making test strips, including nitrocellulose working membrane (CNPC-15), fiberglass conjugate membrane (PT-R7), sample membrane (GFB-R4), and final adsorption membrane (AP045), were manufactured by Advanced Microdevices (Ambala Cantt, India). The 96-well transparent polystyrene microplates Costar 9018 for enzyme-linked immunosorbent assays (ELISA) were purchased from Corning Costar (Tewksbury, MA, USA).

The used strains of microorganisms were obtained from the State Collection of Pathogenic Microorganisms and Cell Cultures «GKPM–OBOLENSK» (Obolensk, Moscow region, Russia): *Salmonella* Typhimurium ATCC 19585, *S.* Enteritidis 3-2, *S.* Paratyphi A56, *S.* Virchow 06, *S.* Anatum 1120, *Escherichia coli* 0157:H7 ATCC51658, *Listeria monocytogenes* NCTC7973, *Yersinia enterocoliytica* H-26-06, *Y. pseudotuberculosis* 4320, *Pseudomonas aeruginosa* ATCC27853, and *Franciella tularensis holarctica* 15NIIEG.

### 2.2. ELISA Testing of Anti-Salmonella Antibodies

The antigen-binding properties of anti-*Salmonella* antibodies were tested by ELISA. All measurements were performed twice. To do this, *S.* Typhimurium cells with concentrations of 10^6^ cells/mL in 50 mM phosphate buffer with 100 mM NaCl, pH 7.4 (PBS) were adsorbed into microplate wells for 1.5 h at 37 °C. After four-fold washing of the wells by PBS with 0.05% Triton X-100 (PBST), the anti-*Salmonella* antibodies were added at concentrations varying from 3 μg/mL to 0.3 ng/mL in PBST and incubated for 45 min at 37 °C. Then, the washing was performed in the same manner, and GAMI–HRP solution (1:5000 in PBST) was added to the wells, incubated for 45 min at 37 °C, and the microplate was washed against 4 times with PBST.

To record the catalytic activity of the bound HRP, its substrate solution—0.42 mM TMB and 1.8 mM H_2_O_2_ in 0.1 M citrate buffer, pH 4.0—was added to the microplate wells. After 15 min of incubation at room temperature, the reaction was stopped by adding H_2_SO_4_; its final concentration in the well was 0.33 M. The absorbance of the obtained oxidized and colored TMB derivative was measured at 450 nm using a Zenyth 3100 microplate vertical photometer (Anthos Labtec Instruments, Wals, Austria).

### 2.3. Biotinylation of Antibodies

The antibodies were biotinylated as described by Hermanson [39]. Their solution (100 μM in PBS, 200 μL) was mixed with a solution of biotin N-hydroxysuccinimide ester (1 mM in DMSO, 6.25 μL) and incubated for 2 h at room temperature and constant stirring. Unreacted low molecular weight compounds were removed by dialysis against PBS.

### 2.4. ELISA Detection of Salmonella Cells

To perform the ELISA of *Salmonella* cells in sandwich format, anti-*Salmonella* antibodies were adsorbed into microplate wells (with a concentration range from 0.25 to 2 μg/mL in PBS) for 1.5 h at 37 °C. Then, cells of *S.* Typhimurium were added to the wells at a concentration range from 10^8^ to 10^3^ cells/mL in PBST and incubated for 45 min at 37 °C. After this, solutions of biotinylated antibodies (from 0.5 to 4 μg/mL in PBST) and STR–HRP conjugate (dilution 1:5000 in PBST) were consistently added and incubated at each stage for 45 min at 37 °C. The ELISA states were interspersed with washing in the same manner as in the above ELISA protocol. Finally, the catalytic activity of the bound HRP was registered as described above. All measurements were performed in triplicate.

### 2.5. Measuring Constants of Antigen-Antibody Reactions

Equilibrium and kinetic constants for the interactions of specific antibodies with *S.* Typhimurium cells were measured using the iMSPR-Pro biosensor (Icluebio, Seoul, South Korea) and CDex100 chip based on the protocol described in [40]. The surface of the CDex100 chip, modified with carboxymethylated dextran, was activated by passing a 1:1 (*v*/*v*) mixture of aqueous solutions of EDC and NHS at concentrations of 0.1 and 0.4 M, respectively, for 240 s at 25 °C. Then, 1E6cc antibodies with a concentration of 70 μg/mL were applied to a 10 mM Na-citrate buffer, pH 5.0, for their immobilization and the creation of the first bound layer on the sensors’ surface. The next preparatory step was passing *S.* Typhimurium through the biosensor’s cell at a concentration of 10^6^ cells/mL in a 10 mM HEPES buffer, pH 7.4, containing 150 mM NaCl, 3 mM EDTA and 0.005% Tween-20. For the following kinetic registration of immune interaction, the three tested monoclonal antibodies were passed through the cell under the same conditions, and unbound antibodies were removed. Finally, the chip was treated with 100 mM glycine-HCl, pH 2.0 as a regenerating reagent. The chip with a surface blocked by ethylamine was used as a reference. The next cycle of measurements began with the application of *S.* Typhimurium cells to covalently attached 1E6cc antibodies. The kinetic measurements were performed in triplicate for antibody concentrations 200, 150, 100, and 50 nM. The BIA evaluation program “1:1 (Langmuir) binding” (Biacore AB, Uppsala, Sweden) was applied to approximate the obtained biosensor dependences and calculate the equilibrium and kinetic constants.

### 2.6. Synthesis of Gold Nanoparticles

In accordance with [41,42], 1.0 mL of a 1% HAuCl_4_ solution was added to 97.8 mL of deionized water, brought to a boil, and 1.2 mL of a 1% sodium citrate solution was added and the mixture was stirred. The reaction mixture was boiled for 30 min, then cooled and stored at 4–6 °C.

### 2.7. Conjugation of Anti-Salmonella Antibodies with GNPs

Before the conjugation, GNPs (OD_520_ = 1.0) were brought to pH 9.0 by adding 0.1 M potassium carbonate, and the preparations of anti-*Salmonella* antibodies 1E6cc, 10D9H and 5D12A (1 mg/mL) were dialyzed against 1000-fold volumes of 10 mM Tris-HCl, pH 9.0. Then, 10 mL of GNPs were added to 44 μL (44 μg) of the antibodies. The mixtures were incubated for 30 min at room temperature and stirred, after which BSA was added to the final concentration of 0.25%.

The resulting conjugates were separated from unreacted components by a 15 min centrifugation at 20,000× *g* and 4 °C on an Allegra 64R centrifuge (F1202 rotor, Beckman Coulter, Brea, CA, USA). After the removal of supernatants, the conjugate pellets were redissolved in 20 mM Tris-HCl, pH 7.6, containing 1.0% BSA, 1.0% sucrose, 0.1% Tween-20, and 0.1% NaN_3_ (TTBSA), and stored at 4–6 °C.

### 2.8. Characteristics of GNPs and Their Conjugates with Antibodies

The absorption spectra were recorded by Libra S80 spectrophotometer (Biochrom; Cambridge, UK) in the wavelength range from 450 to 600 nm.

Transmission electron microscopy (TEM) was realized as described in [43]. The GNPs and their conjugates were applied to meshes with a pore size of 300 mesh, coated with a backing film made of polyvinylformal dissolved in chloroform. The images were taken on a CX-100 electron microscope (Jeol, Tokyo, Japan) at an accelerating voltage of 80 kV and a magnification of 3,300,000. Digital photographs were processed by the Image Tool software, version 3.0 (University of Texas Health Science Center; San Antonio, TX, USA).

The TEM measurements were carried out using equipment from the Shared-Access Equipment Centre “Industrial Biotechnology” at the Research Center of Biotechnology of the Russian Academy of Sciences.

Dynamic light scattering (DLS) was carried out using Zetasizer Nano device (Malvern Panalytical; Malvern, UK). The particle diameter was registered in the range from 0.1 nm to 10 μm at 25 °C for 10 s, at a scattering angle of 173°. Each sample was analyzed in triplicate, and each measurement was implemented in three runs for a duration of 30 s. The obtained data were processed using Malvern Software ver. 7.11.

### 2.9. Manufacturing Test Strips for LFIA

A GAMI solution with a concentration of 0.5 mg/mL was applied to the control zone (CZ) of the working nitrocellulose membrane, and solutions of anti-*Salmonella* antibodies with concentrations from 0.25 to 1.5 mg/mL were applied to the test zone (TZ) of the membrane using an IsoFlow dispenser (Image Technology; Hanover, NH, USA). The diluting buffer was PBS, and the load was 0.12 μL/mm. In the case of format A, the antibody–GNP conjugates (OD_520_ from 1.0 to 6.0) were additionally applied in TTBSA to a 5 mm wide PT-R7 glass fiber membrane; the load was 0.8 μL/mm. The membranes with coated reagents were dried at room temperature for 12 h.

For format A, the final absorbent membrane AP045 and the sample absorbent membrane GFB-R4 were glued to the upper and lower parts of the substrate, respectively, so that they overlapped the upper and lower edges of the working membrane by 2 mm.

For formats B and C, the protective film was removed from the top of the substrate (CZ side) and the final absorbent membrane AP045 was glued onto the substrate so that it overlapped the upper edge of the working membrane by 2 mm. The bottom part of the backing with adhesive tape was cut off.

The obtained multimembrane composites were cut into 3.5 mm wide test strips using an Index Cutter-1 automatic guillotine (A-Point Technologies, Allentown, PA, USA) and stored at 18–22 °C in ziplock bags without direct light.

### 2.10. Implementation of S. Typhimurium LFIAs in Three Formats

Format A: Test strips were dipped into a sample aliquot of 60 μL for 10 min, removed, and then the result was recorded.

Format B: Equal volumes of the tested sample and the antibody–GNP conjugate were mixed and incubated at room temperature for 1–10 min. Then test strips were placed on a horizontal surface, and 6 µL of the reaction mixture was applied on the lower part of the working membrane (at 2–3 mm above the bottom edge). After this, the test strips were dipped in PBST and incubated for 10 min.

Format C: The test strips were sequentially dipped into (i) a sample aliquot of 50 μL, (ii) PBST, (iii) the antibody–GNP conjugate (OD_520_ = 6), and (iv) into PBST again. Each incubation period lasted 5 min.

All measurements were performed in triplicate. After completion of the assays, the test strips were scanned using a CanoScan 9000F scanner (Canon, Tokyo, Japan), and their digital images were processed by TotalLab TL120 software, version 2.01 (Nonlinear Dynamics, Newcastle, UK).

### 2.11. Preparation of Milk Samples for Their Testing by LFIA

Milk with a fat content of 2.5% and 4.0% was purchased in a local supermarket. For the analysis, they were used without dilution or were diluted by PBST immediately before the testing. For milk with a fat content of 2.5%, its mixtures 4:1, 3:2, 2:3, and 1:4 (*v*/*v*) with PBST were prepared to reach fat contents of 2.0, 1.5, 1.0, and 0.5%, respectively. For milk with a fat content of 4.0%, its mixtures with a ratio of 5:3, 1:1, 3:5, 1:3, and 1:7 (*v*/*v*) with PBST were prepared to reach fat contents of 2.5, 2.0, 1.5, 1.0, and 0.5%, respectively. The final preparations were tested by LFIA as described in the Section 2.10. Two preparations with each dilution were prepared from each milk sample, and each preparation was tested twice.

## 3. Results

### 3.1. Characterization of Anti-S. Typhimurium Antibodies

The binding properties of three anti-*Salmonella* antibodies were preliminarily tested using the ELISA technique for immobilized cells of different microorganisms that interacted with the added antibodies at varied concentrations. The binding was characterized in terms of antibodies titers determined as concentrations providing OD_450_ that exceeded the background nonspecific response by its three-fold standard deviations.

There was no binding of any of the three anti-*Salmonella* antibodies with *S.* Paratyphi, *S.* Virchow, *S.* Anatum, *Escherichia coli* 0157:H7, *Listeria monocytogenes*, *Yersinia pseudotuberculosis*, *Y. enterocoliytica*, *Pseudomonas aeruginosa*, and *Franciella tularensis* cells. The clone 1E6cc was specific only to *S.* Typhimurium (titer 5 ng/mL), and clones 10D9H and 5D12A were specific both to *S.* Typhimurium (titers 120 ng/mL and 1 μg/mL, respectively), and *S.* Enteritidis (titers 200 ng/mL and 1 μg/mL, respectively). The corresponding binding curves are given in Figure S1.

Next, ELISA was carried out in a sandwich format, in which complexes of antibodies were immobilized on a microplate surface, and the antigen (*S.* Typhimurium cells), biotinylated antibodies, and peroxidase-labeled streptavidin are formed sequentially. To implement the sandwich ELISA, its conditions were optimized to maximize the sensitivity and values of detected signals without the appearance of non-specific background binding. In accordance with these criteria, the selected concentration for adsorbing antibodies was 1 μg/mL, and for their biotinylated derivatives was 2 μg/mL. Shortening this to a 15 min incubation with the streptavidin–peroxidase conjugate did not lead to deterioration in the detected signals and assay sensitivity.

The following antibody combinations were the most effective in ELISA testing:

− Immobilized 1E6ss clone—biotinylated 1E6ss clone.
− Immobilized 10D9H clone—biotinylated 1E6ss clone.
− Immobilized 5D12A clone—biotinylated 1E6ss clone.

**Figure 2:**
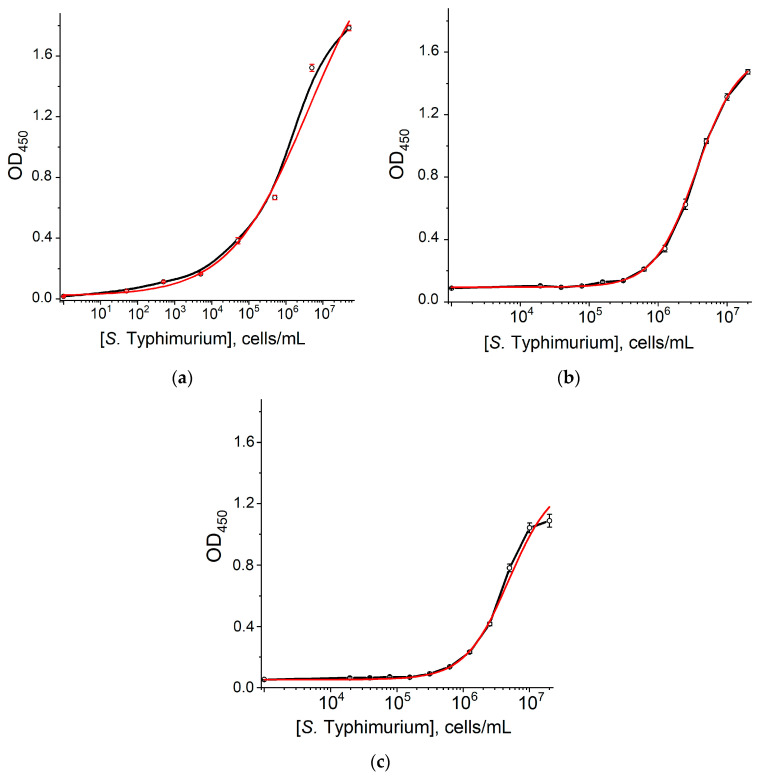
Calibration curves of *S.* Typhimurium in the sandwich ELISA. (**a**)—1E6ss, 1E6ss-biotin; (**b**)—10D9H, 1E6ss–biotin; (**c**)—5D12A, 1E6ss–biotin. Experimental data—black curves, approximation—red curves. All measurements were performed in triplicate.

The corresponding calibration curves are shown in Figure 2. The detection limits of *S.* Typhimurium cells were 3.4 × 10^4^, 5 × 10^5^, and 7 × 10^5^ cells/mL, respectively.

### 3.2. Measurement of Binding Constants for Salmonella Cells–Antibodies Interactions

The anti-*Salmonella* antibodies were also characterized using a surface plasmon resonance biosensor. To prevent the partial destruction of the antigen during the chip’s regeneration for repeated measurements, the earlier proposed approach was used with indirect antigen immobilization on the covalently coupled antibodies and the addition of a new antigen portion after each regeneration cycle [40]. The obtained sensograms for immune interactions (Figure S2) were quantitatively processed to find the parameters of the studied immune interactions.

Considering the descending and ascending parts of the sensograms, we have calculated the following data, summarized in Table 1.

### 3.3. Synthesis and Characterization of Gold Nanoparticles

The spherical GNPs with an expected average diameter of 30–40 nm were synthesized by the Frens method [41] with modifications [42]. The structural characterization of the GNP preparation was carried out by transmission electron microscopy (TEM).

**Figure 3:**
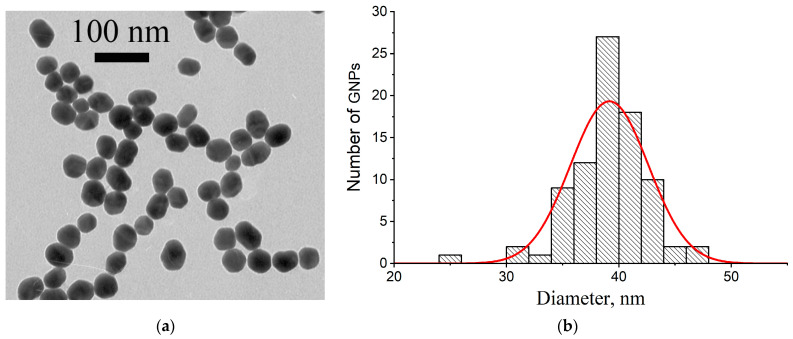
Fragment of electron micrograph for the synthesized GNPs (**a**) and histogram of their distribution by diameter, *n* = 85 (**b**).

As can be seen from Figure 3a with a fragment of an electron micrograph, the GNPs are relatively homogeneous in size and have a near-spherical shape. This visual assessment was confirmed by computer processing of the digital photographs. The histogram of GNPs (from Figure 3a) distribution in their diameters is given in Figure 3b. The average diameter for 85 GNPs was 39.3 ± 3.2 nm. The ellipticity coefficient, calculated as the ratio of the major and minor axes of the particle, was 1.19 ± 0.21, which indicates that the shape of the GNPs is close to spherical.

It is known that spherical GNPs have a characteristic absorption peak at wavelengths of 515–540 nm associated with the surface plasmon resonance [44]. The absorption spectrum of the synthesized GNP preparation is given in Figure S3 and shows a peak at 528.5 nm. The application of the previously established relationship between the average diameter and the absorption peak position for spherical GNPs [45] gives the average diameter of GNPs as equal to 39 nm, being in full accordance with TEM data.

The hydrodynamic parameters of the GNPs were determined by the DLS method (Figure S4). It was found that the GNPs had an average diameter of 42.8 nm. Their polydispersity index (Pdi) was 0.234, reflecting the good homogeneity of the preparation [46].

Thus, the data on dimensional characteristics of the GNP preparation by the three methods are in good agreement with each other (the differences are no more than 9.7%).

### 3.4. Synthesis and Characterization of Conjugates Between GNPs with Antibodies

The obtained GNPs were conjugated with anti-*Salmonella* antibodies by adsorption immobilization. The ratio of the binding compounds was chosen to reach monolayer covering the GNPs surface as it was investigated in [45]. The calculations implemented in accordance with these recommendations led to the choice of an antibodies concentration of 4.4 μg/mL, which was used for the conjugation.

The average diameters (DLS data, see Table S1) of the conjugates compared to unloaded GNPs increased from 5.8 to 6.6 nm. The Pdi of the conjugates was in the range of 0.248–0.265, indicating the stored similarity of GNPs after their modification by the antibodies. Spectrophotometric data for the conjugates (see Table S1) demonstrate shifts by 6.0–7.5 nm of the absorption peaks to longer wavelengths that accord to common situations with the GNPs–protein complexes [47].

### 3.5. Development of S. Typhimurium LFIAs in Three Formats

The synthesized conjugates were used to realize three formats of sandwich LFIA:

(A) All immunoreagents were pre-applied to test strip membranes and the assay was started by immersing the test strip in the sample.
(B) Only reagents were applied in the control and test zones of the test strip, and the antibody conjugate with GNPs was pre-incubated with the sample.
(C) Reagents were applied in the test and control zones of the test strip, and the assay was carried out by sequentially immersing the strip in the sample, washing buffer, conjugate, and washing buffer again.

Format A of sandwich LFIA is a commonly used, having the simplest implementation. However, several earlier publications demonstrated improved sensitivities for formats B [29,48] and C [31,49] relating to different antigen–antibody combinations.

In the given study, the systematic comparison of the three formats and nine (3 × 3) combinations of the first (in test zone) and second (on GNPs) antibodies were implemented. The obtained values of LODs, background, and maximal specific colorations for *S.* Typhimurium cells detecting in the buffer are integrated into Table 2.

As can be seen, three variants based on a combination of 1E6cc clone, both in the test zone and on the GNPs (marked in Table 3 in bold), significantly exceed the others in terms of these parameters, and in 13 variants from the 27 tested, the specific binding is not reliably recorded at all. In addition, formats A, B, and C are successfully implemented for this combination of antibodies. Note that, among the three tested clones, the 1E6cc clone combines a higher ka (commonly with the 10D9H clone) and the lowest kd and KD.

For these three variants (using the 1E6cc clone twice in three LFIA formats), the assay conditions were optimized by varying the concentrations of immunoreagents. To form TZ, the concentration of the 1E6cc clone varied from 0.25 to 1.5 mg/mL. It was shown (Figure S5) that the coloration intensity increased in the concentration range of 0.25–1 mg/mL and remained constant at concentrations above 1 mg/mL. Thus, a concentration of 1 mg/mL was chosen as optimal. For the formation of CZ, the optimal concentration of GAMI was 0.5 mg/mL, which ensured approximately the same intensity of the colorimetric signal in both zones at high cell concentrations. The OD_520_ of the conjugated GNPs varied in the range of 1–6 (Figure S6). In this interval, the signal intensity in TZ increased, but at OD_520_ > 4, nonspecific background coloration was observed, so the optimal value was chosen as OD_520_ = 4. The time of preincubation in format B varied from 1 to 10 min (Figure S7), and the optimal time was 5 min.

**Figure 4:**
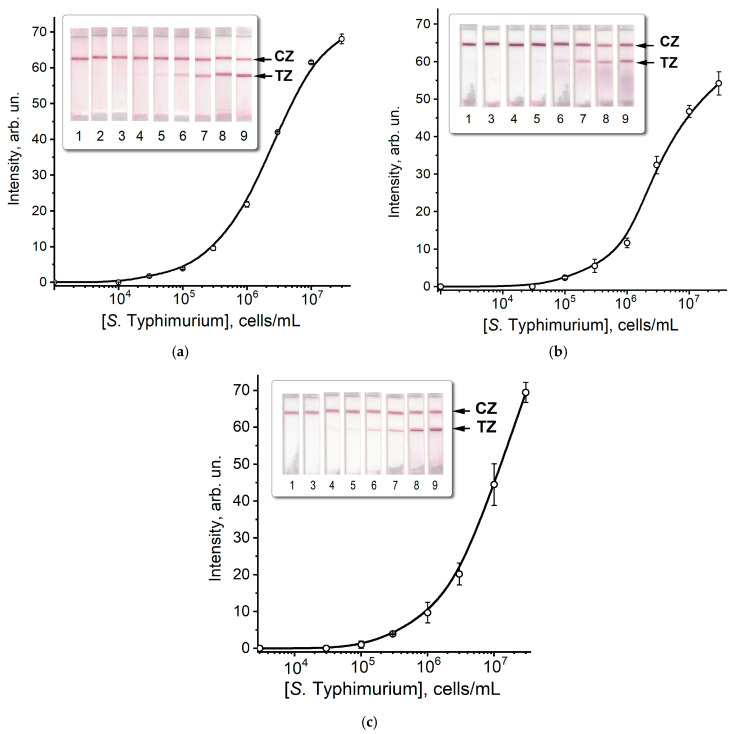
Appearance of test strips and calibration curves of LFIA of *S*. Typhimurium cells of formats A (**a**), B (**b**) and C (**c**). (1–9) correspond to concentrations of 0, 1 × 10^4^, 3 × 10^4^, 1 × 10^5^, 3 × 10^5^, 1 × 10^6^, 3 × 10^6^, 1 × 10^7^, and 3 × 10^7^ cells/mL. CZ—control zone, TZ—test zone. All measurements were performed in triplicate.

Using the selected conditions, the calibration dependence was obtained for the determination of *S.* Typhimurium cells in buffer in three LFIA formats: (Figure 4a–c).

The detection limits of *S.* Typhimurium cells in three LFIA formats were 3 × 10^4^, 1 × 10^5^, and 3 × 10^5^ cells/mL, and the colorations at a cell concentration of 3 × 10^7^ cells/mL were 68, 54, and 69 rel. units and assay time—10, 15, and 20 min, respectively. Thus, the first format with completely applied immunoreagents was optimal. This situation can be interpreted as a result of high affinity in the used antigen–antibody pair that provided efficient binding in the course of the standard lateral flow process with the necessity of enhancing incubations, such as in formats B and C.

### 3.6. LFIA of S. Typhimurium in Cow’s Milk

To validate the proposed test systems, the milk samples were chosen due to the wide contamination of this kind of product by *Salmonella* [50]. Although pasteurization is widely and generally successfully used to kill *Salmonella* in the production of consumer dairy products and, for example, in EU countries, the main health concerns currently arise from *Salmonella* contamination of consumed raw milk [51], for several countries the risks of consuming pasteurized milk contaminated with *Salmonella* remain due to ineffective technological solutions [52,53,54].

To utilize the advantages of LFIA as a rapid testing technique, it should be accomplished with simple and rapid sample preparation. In the case of liquid real samples, such as milk, simple dilution can be successfully applied for this purpose [55,56,57]. However, a high degree of such dilution will worsen the assay sensitivity per g of the initial real sample. Thus, the dilution degree for the tested real samples should be reasonably chosen.

In the present study, commercial milk preparations with 2.5 and 4.0% fat content were used, and diluted with PBST to reach the final fat contents of 3.0, 2.0, 1.5, 1.0, and 0.5%, which were tested by the developed LFIA in three formats. It was found that the accordance with the earlier obtained calibration curves of *S.* Typhimurium detection in buffer was reached for formats A and C when milk is diluted to the fat content of 1.0%, i.e., 2.5 and 4 times (Table S2). In contrast, format B did not give acceptable results even with the matrix effect excluded, including at a dilution of 0.5%. Thus, the dilution of real milk samples with PBST to a fat content of 1.0% was chosen as an efficient recommendation for sample pretreatment, integrating simplicity and rapidity. All measurements were performed twice.

Based on this decision, the three proposed LFIA formats were tested for analyte recovery. Three concentrations of *S.* Typhimurium cells were used, selected from the working range of the calibration curve, namely 5 × 10^5^, 2 × 10^6^, and 5 × 10^6^ cells per 1 g of milk. The results of the recovery testing are summarized in Table 3.

As can be seen, LFIAs according to formats A, B, and C make it possible to detect, respectively, 70–110%, 6–15, and 24–90% of *S.* Typhimurium cells in milk. Regarding the recovery value of 110 ± 8.0% (format A, 2.5% fat content), the absence of its statistically reliable difference from the measurements without a matrix influence can be noted. It follows from the SD of measurements in the buffer for the same concentration of cells that is equal to 2.7%—see the data in Table S3.

Thus, only format A demonstrates acceptable and relatively reproducible recovery values that, in combination with the lowest LOD, cement its choice as a sensitive and efficient technique for *S*. Typhimurium detection in milk. Achieving a low LOD allows for the more effective control of the presence or absence of pathogens in the sample (in accordance with regulatory zero-tolerance requirements). The preliminary stage of microorganisms’ growth can be shortened, and in this way, results of the testing will be obtained faster.

## 4. Conclusions

This investigation comprises a comparative evaluation of three LFIA formats for *Salmonella* Typhimurium, a priority pathogenic contaminant of milk, namely common sandwich LFIA with all immunoreagents pre-applied to the test strip (format A), LFIA with preliminary incubation of the sample and (gold nanoparticle—antibody) conjugate (format B), and LFIA with sequential passages of the sample and the conjugate along the test strip (format C). The assays were realized for different combinations with monoclonal anti-*Salmonella* antibodies and integrated with quantitative characterization of the antigen–antibody interaction. The most sensitive assaying was realized with the use of a high affine 1E6cc clone being applied both for immobilization in the test zone and for conjugation with gold nanoparticle labels. The LFIA conditions were optimized for the chosen reactants, and the detection limits that were reached and testing times for formats A, B, and C were 3 × 10^4^, 1 × 10^5^, and 3 × 10^5^ cells/mL, 10, 15, and 20 min, respectively. The chosen format A in combination with a rapid and simple treatment of samples limited by their dilution demonstrated its efficiency for *Salmonella* cells revealing in milk samples; the recovery values varied in a range from 70 to 110%.

It should be noted that formats B and C, that were proposed in the literature to improve the conditions of immune interactions and, through this, to reach lower detection limits, were found to be inefficient in the studied case. Thus, we can assume that a high affinity of immune interactions provides a sufficient number of the formed immune complexes in the course of a common lateral flow process and excludes the possibility of increasing their number due to an increase in the duration of immune interactions and changes in their conditions. These factors could be important for the further developments of LFIAs for various analytes. Also, the presented design of the study is not associated with specific features of the target analyte and used antibodies, it can be transferred to LFIAs of different bacteria. Concerning pathogens detection in other kinds of foods, especially solid ones, the proposed assay should be integrated with the existing protocols for the preparation of liquid extracts, providing a high degree of transition of bacterial cells into them. To avoid the influence of some compounds of various samples, such as fat, on LFIA (change in the speed of lateral flow, blockage of membrane pores), they may be separated by centrifugation or by the addition of reactants causing specific precipitation.

## Figures and Tables

**Table 1:** Constants for anti-*Salmonella* antibodies interactions with *S.* Typhimurium cells. Abbreviations: ka—average value of kinetic association constant for experiments at different cell concentrations; kd—average value of kinetic dissociation constant for experiments at different cell concentrations; KD—equilibrium dissociation constant defined as the kd/ka ratio.

Antibody Clone	ka, M^−1^s^−1^	kd, s^−1^	KD, M
1E6cc	(5.0 ± 1.7) × 10^4^	(6.2 ± 0.6) × 10^−4^	1.2 × 10^−8^
10D9H	(5.5 ± 0.4) × 10^4^	(2.7 ± 0.3) × 10^−3^	4.9 × 10^−8^
5D12A	(1.5 ± 0.4) × 10^4^	(1.5 ± 0.1) × 10^−3^	1.0 × 10^−7^

**Table 2:** Analytical parameters of LFIA of *S*. Typhimurium cells in formats A, B and C. The colorations were measured at a concentration of *S*. Typhimurium cells equal to 3 × 10^7^ cells/mL.

Antibodies in TZ	1E6cc	1E6cc	1E6cc	10D9H	10D9H	10D9H	5D12A	5D12A	5D12A
Antibodies with GNP	1E6cc	10D9H	5D12A	1E6cc	10D9H	5D12A	1E6cc	10D9H	5D12A
Format A									
LOD, cells/mL	**(9.3 ± 0.8)** × **10^4^**	(7.0 ± 0.9) × 10^6^	(9.2 ± 1.2) × 10^6^	(9.0 ± 1.1) × 10^4^	–	–	(1.8 ± 0.6) × 10^6^	–	–
Coloration of TZ, arb. un.	**68.0 ± 1.4**	6.8 ± 0.8	5.3 ± 0.3	14.8 ± 1.4	–	–	7.4 ± 1.0	–	–
Background coloration, arb. un.	**0**	0	0	0	–	–	0	–	–
Format B									
LOD, cells/mL	**(8.8 ± 0.3)** × **10^5^**	(8.2 ± 0.6) × 10^5^	(2.2 ± 0.5) × 10^6^	(3.3 ± 0.4) × 10^4^	–	–	(1.3 ± 0.8) × 10^5^	–	–
Coloration of TZ, arb. un.	**54.2 ± 3.1**	79.0 ± 9.5	80.8 ± 5.4	26.4 ± 4.2	–	–	43.9 ± 1.1	–	–
Background coloration, arb. un.	**0**	21.5 ± 0.1	3.5 ± 1.0	4.8 ± 0.6	–	–	8.2 ± 0.7	–	–
Format C									
LOD, cells/mL	**(6.5 ± 0.4)** × **10^5^**	–	(9.1 ± 0.8) × 10^5^	(1.3 ± 0.3) × 10^6^	–	–	(7.0 ± 1.2) × 10^5^	–	–
Coloration of TZ, arb. un.	**69.5 ± 2.7**	–	40.1 ± 3.3	63.1 ± 4.7	–	–	54.0 ± 4.3	–	–
Background coloration, arb. un.	**0**	–	0	2.0 ± 0.2	–	–	1.8 ± 0.7	–	–

**Table 3:** Detection of *S.* Typhimurium cells in milk.

Added *S.* Typhimurium, Cells/mL	Revealed *S.* Typhimurium, Cells/mL	Recovery ± SD (%)
Format A		
Milk with 2.5% fat content		
5 × 10^6^	(5.5 ± 0.4) × 10^6^	110 ± 8.0
2 × 10^6^	(1.8 ± 0.1) × 10^6^	90 ± 6.0
5 × 10^5^	(3.8 ± 0.1) × 10^5^	76 ± 1.0
Milk with 4.0% fat content		
5 × 10^6^	(4.8 ± 0.2) × 10^6^	96 ± 4.2
2 × 10^6^	(1.6 ± 0.1) × 10^6^	80 ± 2.5
5 × 10^5^	(3.5 ± 0.1) × 10^5^	70 ± 2.9
Format B		
Milk with 2.5% fat content		
5 × 10^6^	(4.7 ± 0.6) × 10^5^	9.4 ± 1.3
2 × 10^6^	(1.2 ± 0.2) × 10^5^	6.0 ± 1.0
5 × 10^5^	<3 × 10^4^	–
Milk with 4.0% fat content		
5 × 10^6^	(7.5 ± 0.5) × 10^5^	15.3 ± 0.7
2 × 10^6^	(1.4 ± 0.1) × 10^5^	7.0 ± 0.6
5 × 10^5^	(4.5 ± 0.1) × 10^4^	9.0 ± 0.2
Format C		
Milk with 2.5% fat content		
5 × 10^6^	(3.6 ± 0.3) × 10^6^	72 ± 5.1
2 × 10^6^	(1.8 ± 0.1) × 10^6^	90 ± 5.5
5 × 10^5^	(2.5 ± 0.2) × 10^5^	50 ± 3.8
Milk with 4.0% fat content		
5 × 10^6^	(3.4 ± 0.1) × 10^6^	68 ± 0.6
2 × 10^6^	(4.8 ± 0.1) × 10^5^	24 ± 2.1
5 × 10^5^	(1.7 ± 0.1) × 10^5^	34 ± 2.0

## Data Availability

The raw data supporting the conclusions of this article will be made available by the authors on request.

## Supplementary Materials

The following supporting information can be downloaded at: https://www.mdpi.com/article/10.3390/microorganisms12122555/s1. Figure S1: Dependence of the OD_450_ in ELISA on the concentration of anti-*Salmonella* antibodies 1E6cc (1), 10D9H (2) and 5D12A (3) upon immobilizing *S*. Typhimurium (a) and *S*. Enteritidis (b); Figure S2: Sensograms of the *S*. Typhimurium cells interaction with the anti-*Salmonella* antibodies 1E6cc, 10D9H, and 5D12A; Figure S3: Absorption spectrum for GNPs; Figure S4: DLS measurements for GNPs; Figure S5: Dependence of the coloration intensity of the test zone (TZ) on the concentration of 1E6cc antibodies at a concentration of *S*. Typhimurium cells equal to 3 × 10^7^ cells/mL; Figure S6: Dependence of the coloration intensity of the test zone on the OD_520_ of the used dilution of the 1E6cc–GNP conjugate; Figure S7: Dependence of the coloration intensity of the test zone on the time of preincubation in the format B at a concentration of *S*. Typhimurium cells equal to 3 × 10^7^ cells/mL; Table S1: Characterization of 1E6cc–GNP, 10D9H–GNP and 5D12A–GNP conjugates by spectrophotometry and DLS; Table S2: Selection of dilution levels of milk samples for subsequent LFIA testing; Table S3: SD of measurements of *S*. Typhimurium cells in PBST.
