# Supplementary material for: Lateral Flow Immunosensing of Salmonella Typhimurium Cells in Milk: Comparing Three Sequences of Interactions
Source: Microorganisms. 2024 Dec 11;12(12):2555. doi: 10.3390/microorganisms12122555 (PMC11678374; doi:10.3390/microorganisms12122555)
Supplement: Supplementary file 1 [file microorganisms-12-02555-s001.zip › SI-Byzova.pdf]

Supplementary information

Lateral Flow Immunosensing of *Salmonella* Typhimurium Cells in Milk: Comparing Three Sequences of Interactions

Nadezhda A. Byzova [1], Irina V. Safenkova [1], Alexey A. Gorbatov [2], Sergey F. Biketov [2], Boris B. Dzantiev [1] and Anatoly V. Zherdev [1,*]

[1] A.N. Bach Institute of Biochemistry, Research Centre of Biotechnology of the Russian Academy of Sciences, 119071 Moscow, Russia; nbyzova@inbi.ras.ru (N.A.B.); safenkova@inbi.ras.ru (I.V.S.); dzantiev@inbi.ras.ru (B.B.D.)
[2] State Research Center for Applied Microbiology & Biotechnology, 142279 Obolensk, Russia; gorbatov1986@mail.ru (A.A.G.); biketov@mail.ru (S.F.B.)
* Correspondence: zherdev@inbi.ras.ru; Tel.: +7-495-954-2804

Table of Figures and Tables

Fig. S1 Dependence of the OD_{450} in ELISA on the concentration of anti-*Salmonella* antibodies 1E6cc (1), 10D9H (2) and 5D12A (3) upon immobilizing *S.* Typhimurium (a) and *S.* Enteritidis (b)……………………………………………………………………………………………2

Fig. S2 Sensograms of the *S*. Typhimurium cells interaction with the anti-*Salmonella* antibodies 1E6cc, 10D9H, and 5D12A ………………………………………………………………………3

Fig. S3 Absorption spectrum for GNPs (the red arrow shows maximal OD at 528.5 nm)……….4

Fig. S4 DLS measurements for GNPs…………………………………………………………5

Fig. S5 Dependence of the coloration intensity of the test zone (TZ) on the concentration of 1E6cc antibodies at a concentration of *S*. Typhimurium cells equal to 3 × 10^7 cells/mL. All measurements were performed in triplicate……………………………………………………………………6

Fig. S6 Dependence of the coloration intensity of the test zone on the OD_{520} of the used dilution of the 1E6cc–GNP conjugate. Black columns – coloration intensities in the test zone at a concentration of *S*. Typhimurium cells equal to 3 × 10^7 cells/mL, red columns – nonspecific coloration intensities. All measurements were performed in triplicate……………………………7

Fig. S7 Dependence of the coloration intensity of the test zone on the time of preincubation in the format B at a concentration of *S*. Typhimurium cells equal to 3 × 10^7 cells/mL. All measurements were performed in triplicate……………………………………………………………………8

Table S1 Characterization of 1E6cc–GNP, 10D9H–GNP and 5D12A–GNP conjugates by spectrophotometry and DLS……………………………………………………………………9

Table S2 Selection of dilution levels of milk samples for subsequent LFIA testing……………10

Table S3 SD of measurements of *S.* Typhimurium cells in PBST. All measurements were performed in triplicate……………………………..…………………………………………..11

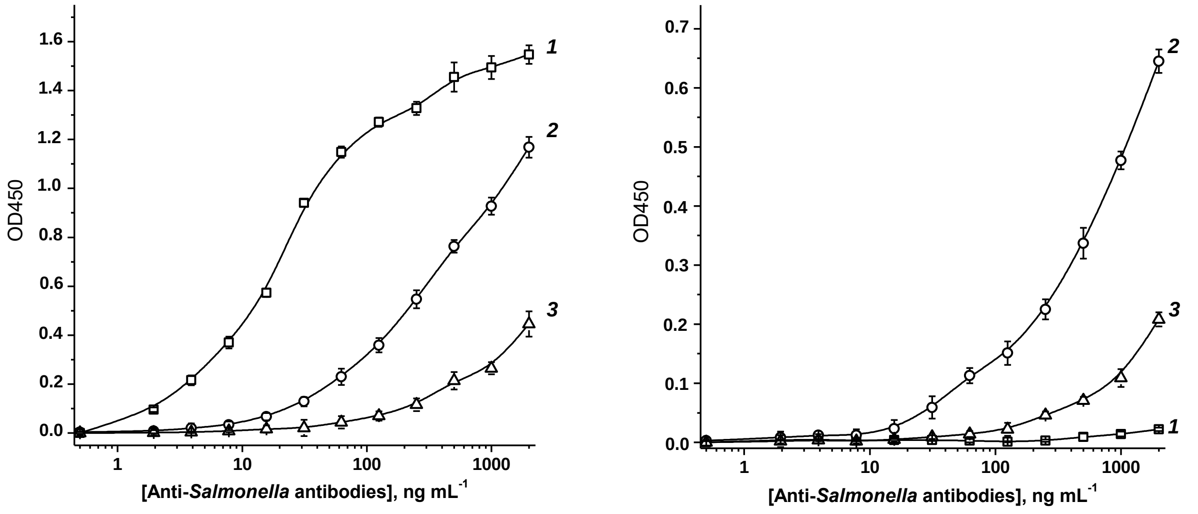

a b

Fig. S1 Dependence of the OD_{450} in ELISA on the concentration of anti-*Salmonella* antibodies 1E6cc (1), 10D9H (2) and 5D12A (3) upon immobilizing *S.* Typhimurium (a) and *S.* Enteritidis (b).

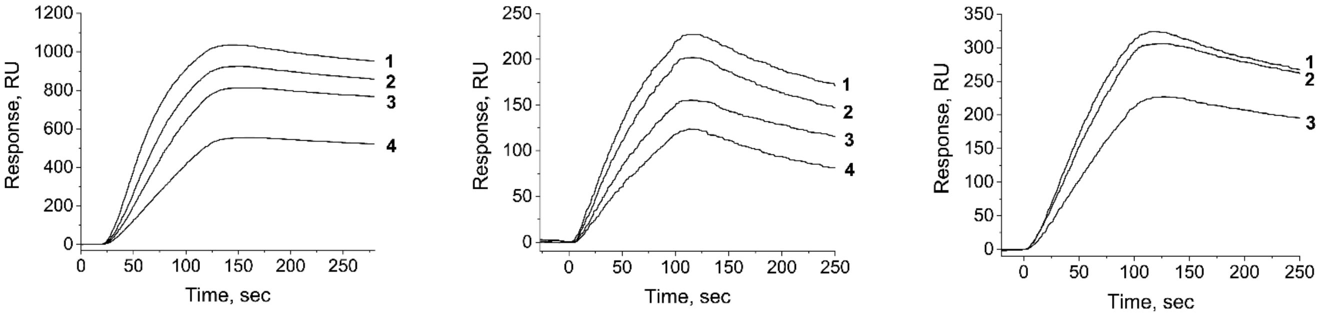

a b c

Fig. S2 Sensograms of the *S. typhimurium* cells interaction with the anti-*Salmonella* antibodies 1E6cc (a), 10D9H (b) and 5D12A (c) at concentrations of 200 (1), 150 (2), 100 (3) and 50 (4) nM.

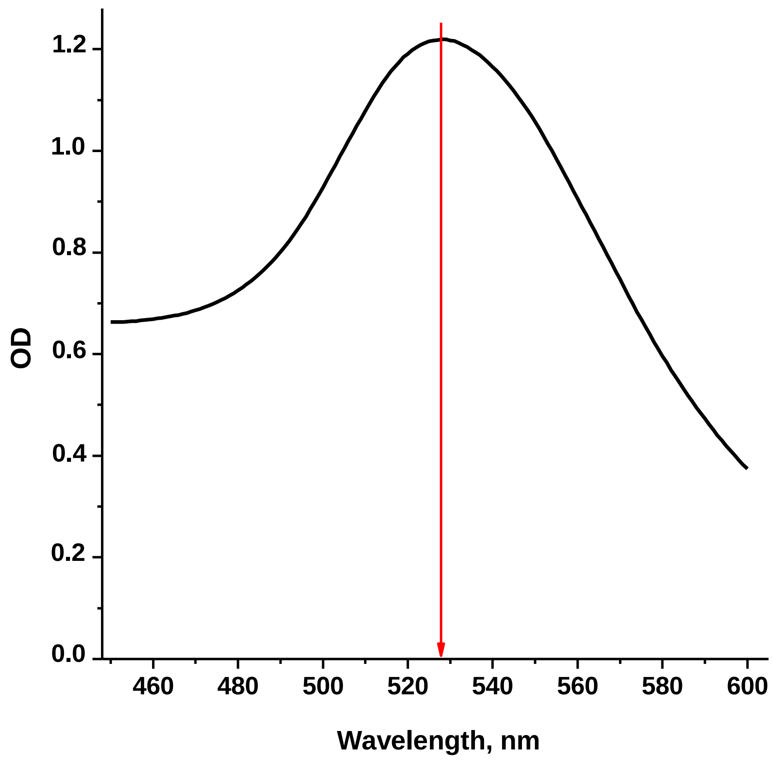

Fig. S3 Absorption spectrum for GNPs (the red arrow shows maximal OD at 528.5 nm).

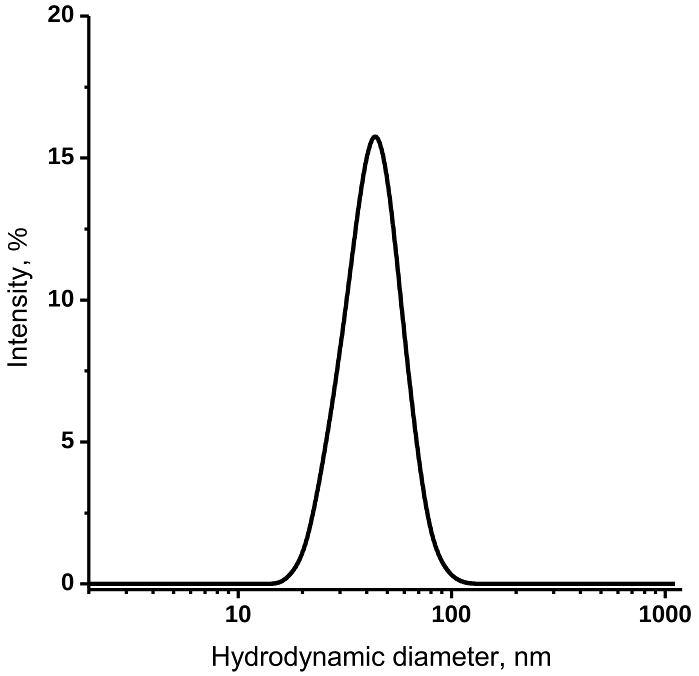

Fig. S4 DLS measurements for GNPs.

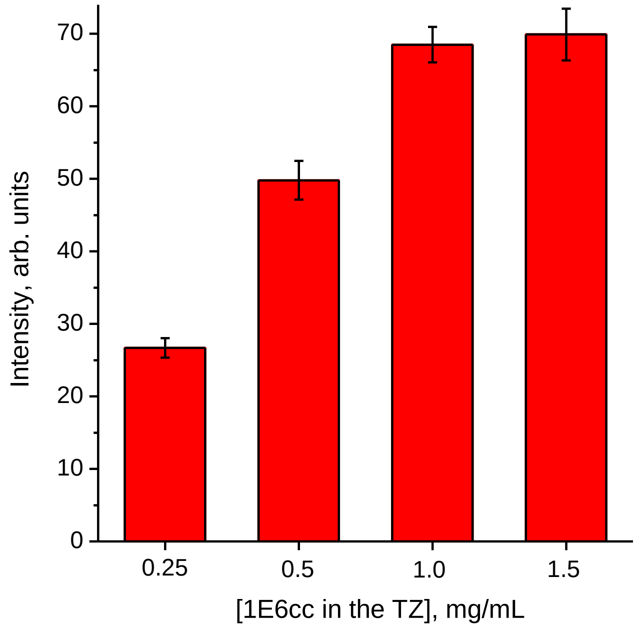

Fig. S5 Dependence of the coloration intensity of the test zone (TZ) on the concentration of 1E6cc antibodies at a concentration of *S*. Typhimurium cells equal to 3×10^7 cells/mL. All measurements were performed in triplicate.

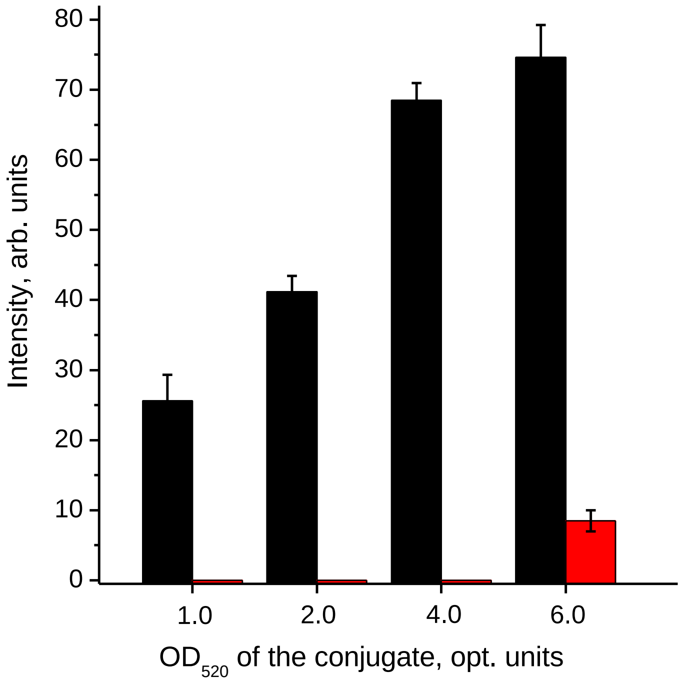

Fig. S6 Dependence of the coloration intensity of the test zone on the OD_{520} of the used dilution of the 1E6cc–GNP conjugate. Black columns – coloration intensities in the test zone at a concentration of *S*. Typhimurium cells equal to 3×10^7 cells/mL, red columns – nonspecific coloration intensities. All measurements were performed in triplicate.

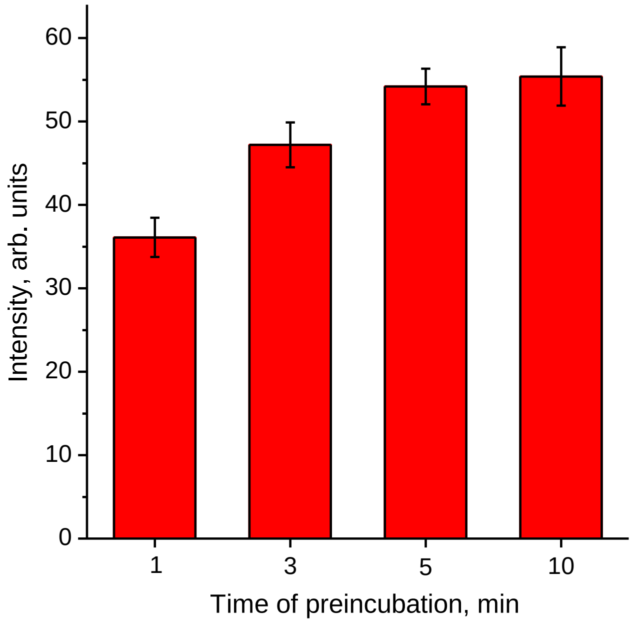

Fig. S7 Dependence of the coloration intensity of the test zone on the time of preincubation in the format B at a concentration of *S*. Typhimurium cells equal to 3 × 10^7 cells/mL. All measurements were performed in triplicate.

Table S1 Characterization of 1E6cc–GNP, 10D9H–GNP and 5D12A–GNP conjugates by spectrophotometry and DLS.

Preparation	Peak wavelength, nm	Diameter (spectral), nm	Diameter (DLS), nm	Pdi (DLS)
GNP	528.5	39.0	42.8	0.234
1E6cc–GNP	531.0	45.0	48.6	0.248
10D9H–GNP	531.5	46.5	49.4	0.265
5D12A–GNP	531.0	45.0	48.8	0.252

Table S2 Selection of dilution levels of milk samples for subsequent LFIA testing.

Parameters	PBST	Final fat content of milk after dilution, %					
**Format A**							
Milk of 2.5% fat content			2.5	2.0	1.5	1.0	0.5
Signal in TZ, arb. un.	68.1±0.7		38.3±1.2	40.9±1.1	56.7±0.8	67.2±0.6	67.4±0.5
LOD, cells/mL	(3.0±0.1) × 10^4		(1.4±0.2) × 10^5	(7.5±0.3) × 10^4	(4.5±0.2) × 10^4	(3.0±0.2) × 10^4	(3.0±0.1) × 10^4
Milk of 4.0% fat content		4.0	3.0	2.0	1.5	1.0	0.5
Signal in TZ, arb. un.	68.1±0.7	41.4±1.2	42.8±1.3	52.5±0.9	60.3±0.8	66.9±0.5	67.1±0.6
LOD, cells/mL	(3.0±0.1) × 10^4	(2.2±0.3) × 10^5	(1.1±0.2) × 10^5	(8.3±0.3) × 10^4	(5.2±0.1) × 10^4	(3.1±0.3) × 10^4	(3.1±0.1) × 10^4
**Format B**							
Milk of 2.5% fat content			2.5	2.0	1.5	1.0	0.5
Signal in TZ, arb. un.	54.0±0.4		–	25.8±1.2	31.3±1.1	44.6±1.1	48.4±0.9
LOD, cells/mL	(1.8±0.4) × 10^5		–	(3.5±0.2) × 10^5	(8.1±0.3) × 10^5	(1.2±0.2) × 10^6	(1.5±0.1) × 10^6
Milk of 4.0% fat content		4.0	3.0	2.0	1.5	1.0	0.5
Signal in TZ, arb. un.	54.0±0.4	–	30.5±1.5	36.3±1.2	39.2±1.3	46.2±1.4	49.0±0.8
LOD, cells/mL	(1.8±0.4) × 10^5	–	(3.3±0.2) × 10^5	(6.2±0.3) · 10^5	(8.5±0.3) × 10^5	(1.1±0.2) × 10^6	(2.2±0.3) × 10^6
**Format C**							
Milk of 2.5% fat content			2.5	2.0	1.5	1.0	0.5
Signal in TZ, arb. un.	69.2±0.5		46.1±1.2	50.5±1.2	61.6±1.0	68.3±0.8	68.5±0.7
LOD, cells/mL	(3.1±0.1) × 10^5		(2.2±0.3) × 10^6	(8.3±0.4) × 10^5	(5.5±0.1) × 10^5	(3.1±0.2) × 10^5	(3.0±0.1) × 10^5
Milk of 4.0% fat content		4.0	3.0	2.0	1.5	1.0	0.5
Signal in TZ, arb. un.	69.2±0.5	42.8±1.2	45.5±1.1	47.8±0.8	54.4±0.8	68.6±0.6	68.7±0.6
LOD, cells/mL	(3.1±0.1) × 10^5	(3.2±0.3) × 10^6	(1.4±0.2) × 10^6	(6.8±0.5) × 10^5	(5.4±0.2) × 10^5	(3.0±0.1) × 10^5	(3.0±0.1) × 10^5

Table S3 SD of measurements of *S.* Typhimurium cells in PBST. All measurements were performed in triplicate.

**Format of LFIA**	**Added *S.* Typhimurium, cells/mL**	**± SD (%)**
**A**		
	5×10^6	± 2.7
	2×10^6	± 3.8
	5×10^5	± 3.5
**B**		
	5×10^6	± 4.2
	2×10^6	± 6.3
	5×10^5	± 9.6
**C**		
	5×10^6	± 3.3
	2×10^6	± 4.6
	5×10^5	± 4.7
